# Analysis of Recombinant Cedar Virus Infection and Cross-Protection Against Related Henipaviruses in African Green Monkeys

**DOI:** 10.3390/v18030292

**Published:** 2026-02-28

**Authors:** Declan D. Pigeaud, Moushimi Amaya, Viktoriya Borisevich, Karla A. Fenton, Krystle N. Agans, Courtney Woolsey, Antony S. Dimitrov, Abhishek N. Prasad, Natalie S. Dobias, Daniel J. Deer, Joan B. Geisbert, Robert W. Cross, Christopher C. Broder, Thomas W. Geisbert

**Affiliations:** 1 Galveston National Laboratory, University of Texas Medical Branch, Galveston, TX 77555, USAviborise@utmb.edu (V.B.); kafenton@utmb.edu (K.A.F.); knagans@utmb.edu (K.N.A.); cbwillia@utmb.edu (C.W.); abnprasa@utmb.edu (A.N.P.); nsdobias@utmb.edu (N.S.D.); djdeer@utmb.edu (D.J.D.); jbgeisbe@utmb.edu (J.B.G.); rwcross@utmb.edu (R.W.C.); 2 Department of Microbiology and Immunology, University of Texas Medical Branch, Galveston, TX 77555, USA; 3 Department of Microbiology and Immunology, Uniformed Services University, Bethesda, MD 20814, USA; moushimi.amaya@usuhs.edu (M.A.); antony.dimitrov.ctr@usuhs.edu (A.S.D.); 4 Henry M. Jackson Foundation for the Advancement of Military Medicine Inc., Bethesda, MD 20814, USA

**Keywords:** Cedar virus, Nipah virus, Hendra virus, nonhuman primate, intratracheal route, intranasal route, pathogenesis, animal model, cross-protection

## Abstract

Cedar virus (CedV), related to the highly pathogenic bat-borne henipaviruses, Hendra virus (HeV) and Nipah virus (NiV), is non-pathogenic in small animal models, likely due to the inability to produce interferon-antagonist proteins. We evaluated the pathogenesis of recombinant CedV (rCedV) in the African green monkey (AGM) model and determined if prior infection conferred cross-protective immunity against a lethal challenge with NiV Bangladesh (NiV-B) or HeV. AGMs infected with rCedV remained asymptomatic, with no clinical signs of disease or detectable viremia. The rCedV infected animals developed homologous neutralizing antibody responses that failed to cross-neutralize NiV-B or HeV. At 42 days post-rCedV infection, AGMs were challenged with a lethal dose of NiV-B or HeV, and prior infection with rCedV failed to protect against NiV-B challenge, with all animals succumbing to NiV-B. Similarly, rCedV infection did not confer consistent protection against HeV, with 2/4 animals succumbing to lethal HeV. These findings confirm that CedV is non-pathogenic in the AGM model of NiV and HeV infection, justifying its classification as a BSL-2 agent. The findings also demonstrate that rCedV does not elicit a cross-protective immune response to prevent lethal disease from either NiV-B or HeV highlighting significant immunological differences between CedV and the pathogenic henipaviruses.

## 1. Introduction

Hendra virus (HeV) and Nipah virus (NiV), prototypical members of the Henipavirus genus within the Paramyxoviridae family [1], pose significant threats to animal and human health. These viruses are classified as Biosafety Level 4 (BSL-4) pathogens because of the high mortality associated with severe and often fatal respiratory and/or neurological disease in the infected host as well as the lack of vaccines or antivirals approved for human use. Since its emergence in 1994, HeV has caused disease in 110 fatal or euthanized equine cases [2,3,4] and seven human cases of which four were lethal [5]. In 2021, a new strain, HeV genotype 2 (HeV-g2) sharing ~83% whole genome nucleotide identity with the prototype HeV, was discovered in Australia in horses and flying foxes [6,7]. Retrospective analysis identified HeV-g2 as being the causative agent for 2 of the recorded 110 equine cases, 1 in 2015 [6] and 1 in 2021 [8]. NiV emerged in 1998–99 in an outbreak of a respiratory disease in pigs and acute and febrile encephalitis in pig farmers in Malaysia and Singapore [9,10]. In 2001, a genetically distinct strain, NiV-Bangladesh (NiV-B), emerged that exhibited ~92% nucleotide identity across the genome with NiV-Malaysia (NiV-M) [10,11]. NiV-B has since caused nearly annual outbreaks with the most notable outbreak in 2018 in Kerala, India that had a case fatality rate of 91% [12]. NiV-M was also implicated in a 2014 outbreak of encephalitis in horses and humans in the Philippines [13].

The Henipavirus genus has since expanded to include Cedar virus (CedV) [14], Gamak virus, Daeryong virus [15], Langya virus [16], and Salt Gully virus [17] for which viral isolates exist. In addition, the Henipavirus also include Ghana bat virus (GhV) [18], Mojiang virus (MojV) [19], Angavokely virus (AngV) [20], Melian virus, Denwin virus [21] and Ninorex virus [22] but known only from sequence data. Pteropod fruit bats are the natural reservoirs for HeV, NiV, CedV, GhV and AngV [23,24,25,26], while the other henipaviruses are apparently shrew-borne [15,16,19,21,22]. Transmission from bats to well-established spillover hosts such as horses (HeV) or pigs (NiV) occur through the consumption of bat-contaminated feed or fruit, respectively [27,28]. Transmission of NiV to other species including cats, dogs and horses has occurred (Malaysia and the Philippines) as have HeV infection of dogs in Australia. Human infection with HeV or NiV-M results from close or direct contact with infected horses or pigs, respectively [9,29,30]. NiV-B however can directly infect humans through consumption of contaminated date palm sap [31,32,33]. Human-to-human NiV-B transmission through close contact with infected patients have been recorded up to five generations and the apparent NiV-M spillover in the Philippines also involved human-to-human transmission [13,34,35,36,37,38].

CedV was isolated in 2012 in Queensland, Australia from pooled flying fox urine samples [14]. Molecular and phenotypic characterization has demonstrated important differences in CedV entry receptor usage and genetic differences in the phosphoprotein (P) gene when compared to HeV and NiV. HeV and NiV utilize the two highly conserved ephrin proteins (ephrin-B2 and ephrin-B3) as entry receptors [39,40,41,42] demonstrating a possible link to the observed respiratory and neuronal tropism observed during infection. Although CedV utilizes ephrin-B2 it does not use ephrin-B3, and instead uses additional ephrins-B1, -A2 and -A5 [43]. In addition, experimental infection with CedV does not result in any clinical disease in several animal models such as guinea pigs, ferrets, Balb-C mice [14], hamsters [44], albino C57BL/6 mice and IFNAR-deficient [45], or STAT1-deficient mice [46]; a phenotype related to differences in the P gene. The P gene of HeV and NiV contains an RNA editing sequence that allows for the addition of non-templated G bases to produce the V and W nonstructural proteins that are key interferon antagonists contributing to viral pathogenicity (reviewed in [47,48]). Currently, CedV is the only known member that is distinct in the P gene. The CedV P gene lacks RNA editing capability as well as an open reading frame encoding the V and W proteins [14]. Hamster and ferret studies with recombinant NiV variants have demonstrated distinct pathogenic roles of the V and W proteins, where a lack of the V protein resulted in nonlethal infections [49,50,51].

Guinea pigs, Syrian hamsters, cats, ferrets, and African green monkeys (AGMs) have been established as animal models to investigate HeV and NiV pathogenesis and transmission, and to test and evaluate countermeasures to henipavirus infection and disease [52,53,54]. Here, we investigated the pathogenic potential of CedV in AGMs, the gold-standard animal model of NiV and HeV pathogenesis. Two individual experiments were conducted within one cohort of animals, to first assess whether infection with recombinant, wild-type Cedar virus (rCedV) resulted in any pathologies characteristic of NiV and HeV disease and a subsequent study to determine whether prior rCedV infection could protect from lethal disease following experimental challenge with NiV-B or HeV. Together, these studies demonstrate that rCedV infection (i). does not result in clinical disease characteristic of highly pathogenic henipaviruses and (ii). fails to provide any cross-protection in AGMs against either NiV-B or HeV challenge.

## 2. Materials and Methods

### 2.1. Ethics Statement and Study Approval

African green monkeys (AGMs) were maintained in animal BSL-4 containment in the Galveston National Laboratory at the University of Texas Medical Branch (UTMB), Galveston, TX, USA. This facility is assured by the Office of Laboratory Welfare and fully accredited by the Association for Assessment and Accreditation of Laboratory Animal Care International. All research was approved by the UTMB IACUC and complied with the Animal Welfare Act and other federal statutes and regulations pertaining to animal experimentation.

### 2.2. Cells and Challenge Viruses

We previously reported on the construction and rescue of replication-competent, recombinant CedV (rCedV) using reverse genetics [55]. The design of the rCedV antigenome plasmid was based on the Genbank sequence accession no. NC_025351.1.

The NiV-B challenge material employed in this study originated from a fatal human case during an outbreak in Rajbari, Bangladesh in 2004. The challenge material was passaged twice on Vero E6 cells (American Type Culture Collection [ATCC], Manassas, VA, USA #CRL-1586) and the collected supernatants were stored at –80 °C as 1 mL aliquots. Four distinct mutations of sufficient frequency were found between the P2 stock of NiV-B and the reference genome (GenBank AY988601.1). One mutation was noncoding whereas the remaining mutations encode for 3 single amino acid changes: 1 in the M protein and 2 in the F protein [56].

The HeV-prototype isolate used in these studies (GenBank Accession number NC_001906) was obtained from a fatal equine case from the 1994 outbreak in Australia and was provided by Dr. Thomas Ksiazek [57]. All studies with authentic NiV-B and HeV were performed within the BSL-4 facilities of the Galveston National Laboratory, The University of Texas Medical Branch at Galveston, TX, USA.

Vero76 (ATCC #CRL-1587) cells were maintained at 37 °C, 5% CO_2_. Unless otherwise stated all incubations were at 37 °C, 5% CO_2_.

### 2.3. Animal Studies

Animal studies were performed in BSL-4 biocontainment at the Galveston National Laboratory at the University of Texas Medical Branch (UTMB) at Galveston and were approved by the UTMB Institutional Animal Care and Use Committee.

Fourteen henipavirus-seronegative adult AGMs (*Chlorocebus aethiops*, PreLabs, Lehigh Acres, FL, USA) weighing ~5–7 kg were used for these studies. In the first study, eight of these animals, four females and four males, were infected with rCedV with a dose of 5.0 × 10^5^ PFU by the combined intratracheal/intranasal (it/in) route (dose divided equally by route). On day 42 post-rCedV infection 4 AGMs (AGM-1–4) were challenged with NiV-B and 4 AGMs (AGM-5–8) were challenged with HeV. One of the two remaining uninfected rCedV controls (male) was challenged with NiV-B (AGM-9) and the other (female) challenged with HeV (AGM-10). Animals were exposed to NiV-B or HeV via the combined intra-tracheal/intranasal route of infection with 2.5 × 10^5^ PFU was delivered to the nares, divided, and the remaining 2.5 × 10^5^ PFU was delivered directly into the trachea for a total dose of 5.0 × 10^5^ PFU. Following NiV-B or HeV challenge, all animals were closely monitored for evidence of clinical disease and were evaluated utilizing comprehensive numerical clinical scoring criteria based on categories of respiration, appetite, activity/appearance, and neurological signs. Subjects that received a clinical score ≥ 9 were euthanized per institutional protocols.

In addition, clinical pathology assays for hematology and serum biochemistry were performed at days 4, 5, 6, 7, 10, 14, 21, and 28 following NiV-B and HeV challenge. On days 0, 4, 7, 10, 14, 21 and 28 post NiV-B and HeV challenge, plasma, blood and swabs (oral and nasal) were collected for analysis of NiV-B and HeV viral RNA (vRNA) by qRT-PCR.

In the second study, 4 additional AGMs (PreLabs, Lehigh Acres, FL, USA) weighing ~4–6 kg (AGM-11–14) were infected with rCedV with a dose of 5.0 × 10^5^ PFU by the combined it/in route and were euthanized at study day 28. Following rCedV challenge, all animals were closely monitored for evidence of clinical illness (respiration, changes in behavior and reduced activity) and a complete blood count and blood chemistry were performed at days 1, 3, 5, 7, 10, 14, 21 and 28. At the end of the study, day 28, various tissues were collected and analyzed for rCedV vRNA by qRT-PCR.

### 2.4. Clinical Pathology

Total white blood cell (WBC) counts, platelet (PLT), neutrophils (NEU) and red blood cell (RBC) counts were determined from EDTA-blood with the Vetscan HM5 laser-based hematological analyzer (Zoetis, Parsippany, NJ, USA). Serum samples were tested for concentrations of blood urea nitrogen (BUN), glucose (GLU), creatinine (CRE), albumin (ALB), calcium (CA), amylase (AMY), alanine aminotransferase (ALT), aspartate aminotransferase (AST), alkaline phosphatase (ALP), C-reactive protein (CRP), gamma-glutamyltransferase (GGT) and total protein (TP) using a Piccolo point-of-care analyzer and Biochemistry Panel Plus analyzer disks (Zoetis, Parsippany, NJ, USA).

### 2.5. Virus Titrations

Titers of infectious rCedV, NiV-B, and HeV were detected in plasma specimens by standardized plaque assay. Briefly, plasma samples collected by venipuncture were serially diluted in Eagle’s Minimal Essential Medium (EMEM) supplemented with 2% heat inactivated fetal bovine serum (HI-FBS) (E2) and adsorbed in duplicate on confluent Vero76 (ATCC #CRL-1587) cell monolayers in 6-well plates for 1 h before being overlayed with 0.8% agarose in 2X MEM containing 5% HI-FBS and returned to incubate for 2 (HeV) or 3–4 (NiV-B, rCedV) days. A 5% neutral red solution prepared in sterile PBS supplemented with 5% HI-FBS was added to each well and incubated for approximately 24 h after which the stain was decanted and plaques were quantified as plaque-forming units (PFU) per mL (PFU/mL).

### 2.6. Quantification of Viral Genomes

HeV or NiV-B viral RNA from whole blood or tissue specimens was performed by quantitative reverse-transcriptase polymerase chain reaction (qRT-PCR), as described previously [58]. The primer sequences used for quantification of rCedV vRNA were obtained from Marsh et al., 2012 [14] and were optimized for assay specific parameters.

### 2.7. Plaque Reduction Neutralization Test (PRNT)

Neutralizing antibody titers were measured by conventional serum neutralization assays. Briefly, sera serially diluted two-fold in E2 were incubated with ~100 PFU of rCedV, NiV-B or HeV for 1 h. A standardized plaque assay was performed for virus-serum mixtures as described in the virus titrations section. The 50% neutralization titer was determined as the serum dilution at which there was a 50% reduction in plaque counts versus untreated control wells.

### 2.8. Histopathology and Immunohistochemistry

Necropsy was performed on all subjects. Tissue samples of all major organs were collected and immediately fixed in 10% neutral buffered formalin for histopathologic and immunohistochemical (IHC) examination. Tissue sections were deparaffinized and rehydrated through xylene and graded ethanols. Slides went through heat antigen retrieval in a steamer at 95 °C for 20 min in Sigma Citrate Buffer, pH6.0, 10× (Sigma Aldrich, St. Louis, MO, USA). The tissue sections were processed for IHC using the Thermo Autostainer 360 (ThermoFisher, Kalamazoo, MI, USA). Immunoreactivity was detected using an anti-Henipavirus N primary antibody at a 1:4000 dilution for 60 min. The secondary antibody used was biotinylated goat anti-rabbit IgG (Vector Laboratories, Newark, CA, USA #BA-1000) at 1:200 for 30 min followed by Streptavidin Alkaline Phosphatase at a dilution of 1:200 for 20 min (Vector Laboratories, Newark, CA, USA #SA-5100). Slides were developed with Bio-Red (Biopath Laboratories, Oklahoma City, OK, USA #BP-100-FR) for 7 min and counterstained with hematoxylin for 45 s.

## 3. Results

### 3.1. Experimental Infection of AGMs with Recombinant Cedar Virus (rCedV)

**Figure 1:**
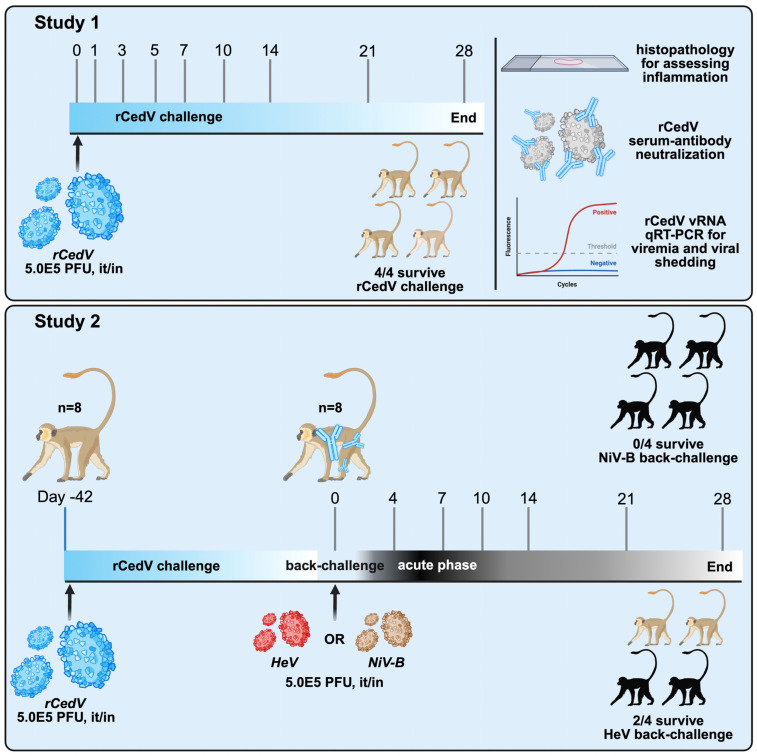
Study design of rCedV infection and back-challenge experiments. In study 1, n = 4 AGMs received a 5.0 × 10^5^ PFU it/in dose of rCedV and blood samples were collected on days 1, 3, 5, 7, 10, 14, 21 and 28. At the end of the study, day 28, various tissues were collected and analyzed for rCedV RNA. In study 2, 8 of 10 AGMs received a 5.0 × 10^5^ PFU dose of rCedV by the combined intratracheal/intranasal (it/in) route. On day 42, the rCedV infected AGMs were challenged with a 5.0 × 10^5^ PFU it/in dose of either NiV-B (n = 4) or HeV (n = 4). The two remaining AGMs served as in-study positive controls for NiV-B or HeV (n = 1 AGM each). Blood samples were collected on days 0, 4, 7, 10, 14, 21 and 28 following NiV-B and HeV challenge. Icons with the illustration were obtained from NIAID NIH BioArt Source and BioRender. Created in BioRender. Declan Pigeaud. (2026) https://BioRender.com/4iwxjul, accessed on 8 February 2026.

**Figure 2:**
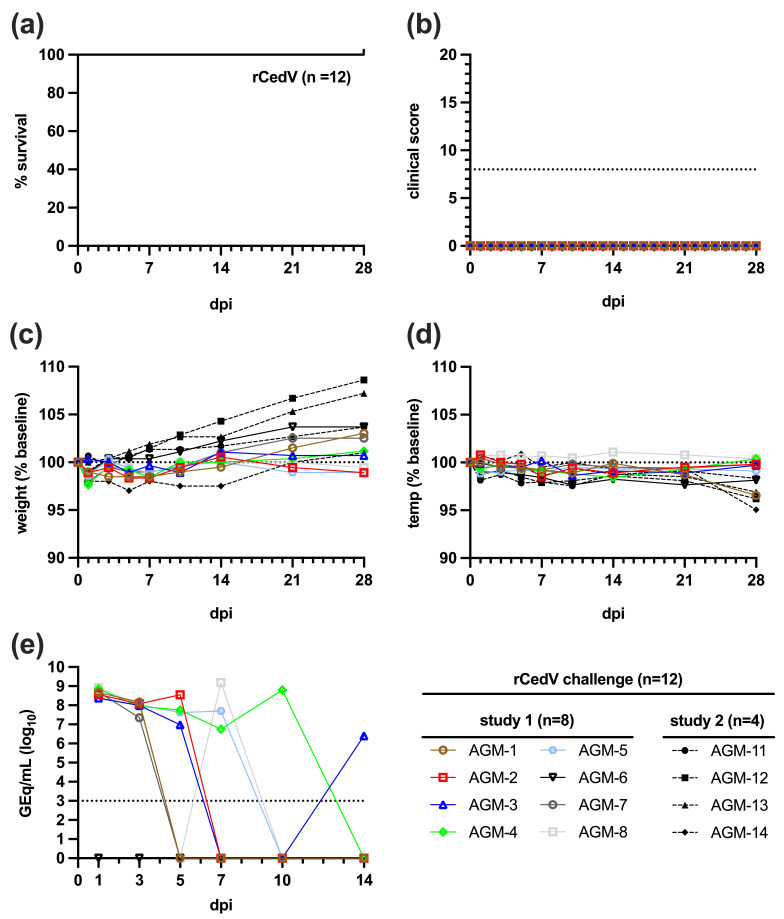
Survival, clinical disease, and antibody response to rCedV infection in healthy AGMs. (**a**) Kaplan–Meier survival curve of n = 12 AGMs challenged with rCedV. (**b**) Comprehensive numerical clinical scores of AGMs challenged with rCedV. Dashed line represents numerical score requiring euthanasia. (**c**) Percent change in weight from baseline (0 dpi) until study day 28. (**d**) Percent change in temperature from baseline until study day 28. (**e**) qRT-PCR detection of rCedV vRNA in nasal swabs collected from AGMs following intranasal infection of rCedV. Individual points represent the mean of two replicates. Dotted line indicates the assay LOD (1000 GEq/mL). Samples with values below the LOD are plotted with a value of 1 to remain visible on the logarithmic scale. vRNA, viral RNA; LOD, limit of detection; dpi, days post-infection.

The design of the studies is shown in Figure 1. Following rCedV challenge all subjects (AGMs 1–8 and 11–14) survived to day 28 of the study and did not show any signs of clinical illness or notable changes in body weight or temperature (Figure 2a–d). Clinical findings for AGMs infected with rCedV are presented in Supplementary Table S1. Hematological analysis and clinical chemistries on whole blood of rCedV infected AGMs performed at the indicated days post-infection (dpi) did not differ significantly from baseline values (Supplementary Figure S1, Supplementary Table S1).

Following infection with rCedV, samples including swabs (oral, nasal, rectal), whole blood, and tissues were assayed by qRT-PCR and plaque assay to quantify rCedV vRNA or infectious virus, respectively. No circulating viremia could be detected as determined by qRT-PCR or plaque assay and no rCedV vRNA could be detected in the tissues of infected animals by qRT-PCR. However, 7 out of 8 (7/8) AGMs had detectable rCedV vRNA in nasal swab samples at several timepoints following challenge but this was likely due to residual inoculum following intranasal challenge (Figure 2e).

### 3.2. Homologous Neutralizing Antibody Response to rCedV Infection

**Figure 3:**
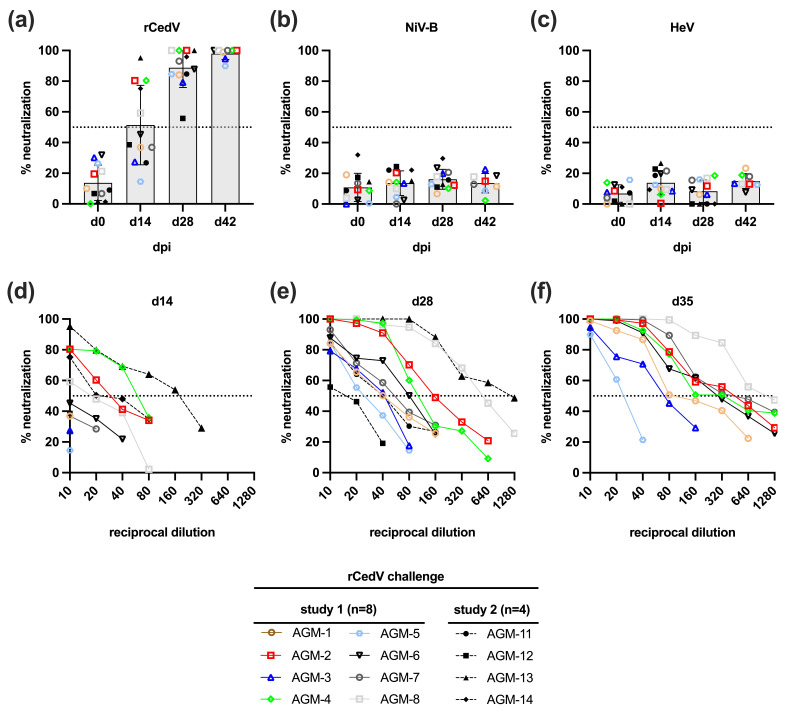
Neutralizing activity of sera from AGMs infected with rCedV in study 1 and 2 by plaque reduction neutralization test (PRNT). Percent neutralization of a 1:10 dilution of serum from specified timepoints against rCedV (**a**), NiV-B (**b**) or HeV (**c**). (**d**–**f**) Percent neutralization curves of serum sampled on days 14, 28, and 35 post-rCedV infection. Individual points represent the mean of two technical replicates, and the data shown are the percent reductions in rCedV plaque counts after incubation with the indicated dilution of sera compared with a control plate (no sera). Horizontal dotted lines indicate 50% neutralization. Individual points for subjects in all panels (**a**–**f**) are formatted according to the figure legend.

We analyzed sera from all rCedV infected AGMs for the presence of anti-CedV neutralizing antibodies by a 50% plaque reduction neutralization test (PRNT_50_) (Figure 3a). Specifically, sera were collected on days 0, 14 and 28 (AGMs 1–8 and 11–14) and on day 42 post-rCedV infection (AGMs 1–8). By day 14, sera from 5/8 rCedV infected AGMs (AGMs 2, 4, 8, 13 and 14) neutralized at least 50% of rCedV titers compared with the virus control plate. By day 28, all AGMs seroconverted with at least 55% neutralizing activity and by day 42, AGMs 1–8 displayed at least 90% neutralizing activity against rCedV. End-point neutralizing antibody titers (expressed as the reciprocal of the last dilution at which at least 50% neutralization was observed) ranged from 20 to 320 on day 28 and from 20 to 640 on day 42 (Figure 3a). Serum samples were also assessed for the neutralization of NiV-B and HeV-prototype, and no meaningful neutralizing activity was identified (Figure 3b,c).

### 3.3. Gross and Histopathological Findings of AGMs Challenged with rCedV

**Figure 4:**
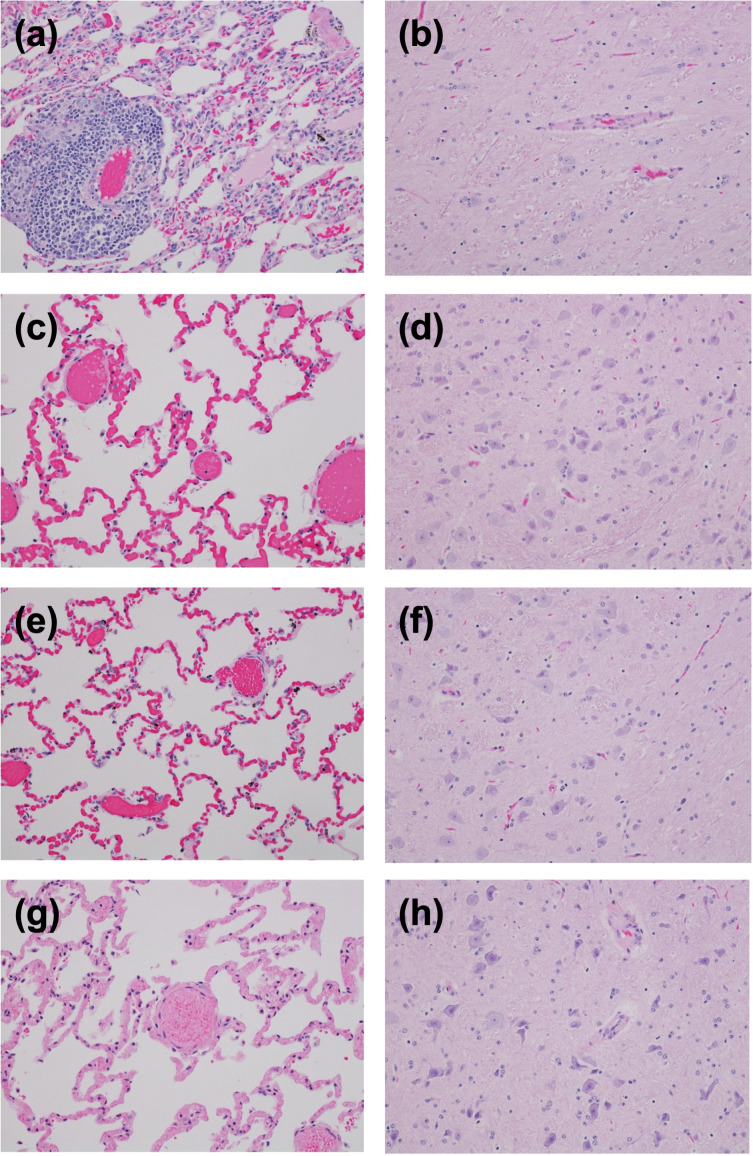
Representative photomicrographs of tissues collected from AGMs infected with recombinant Cedar virus including the lung and brain. Tissues were collected at 28 days post-infection. (**a**) Multifocal lymphohistiocytic perivascular infiltrates with occasional multinucleated giant cells are noted throughout the lung section of AGM-13. In some regions, alveolar septa are minimally expanded with a mixed mononuclear cellular infiltrate. No remarkable inflammatory lesions were noted in the remaining 3 AGMs, AGM-11, AGM-12, and AGM-14 (images, (**c**), (**e**), and (**g**) respectively). All of the examined sections of brainstem had minimal gliosis (images (**b**,**d**,**f**,**h**)).

Microscopic evaluation of tissues (lung and brain) collected from AGMs infected with rCedV from study 2 are shown in Figure 4. The examined sections of lung and brain from this study lacked histologic evidence of overt henipavirus infection, which would include widespread vasculitis, multinucleated syncytia cells of the endothelium, and thrombosis. Mononuclear infiltrates in the lung were noted in a single AGM, AGM-13. Multifocal lymphohistiocytic perivascular infiltrates with occasional multinucleated giant cells are noted throughout the lung section of AGM-13 (Figure 4a). In some regions, alveolar septa are minimally expanded with a mixed mononuclear cellular infiltrate. No remarkable inflammatory lesions were noted in the remaining 3 AGMs, AGMs 11, 12, and 14 (Figure 4c,e,g, respectively). All of the examined sections of brainstem had minimal gliosis (Figure 4b,d,f,h). The pulmonary perivascular infiltrates noted in AGM-13 were not considered classic for henipavirus as it lacked the hallmark lesions, namely endothelial syncytia. The reported minimal gliosis was nonspecific and was also not considered classic for henipavirus. Sections with identified lesions were subjected to IHC staining for anti-henipavirus N antigen; however, no positive staining was identified.

### 3.4. Survival and Clinical Disease of rCedV Infected AGMs Back-Challenged with NiV-B or HeV

**Figure 5:**
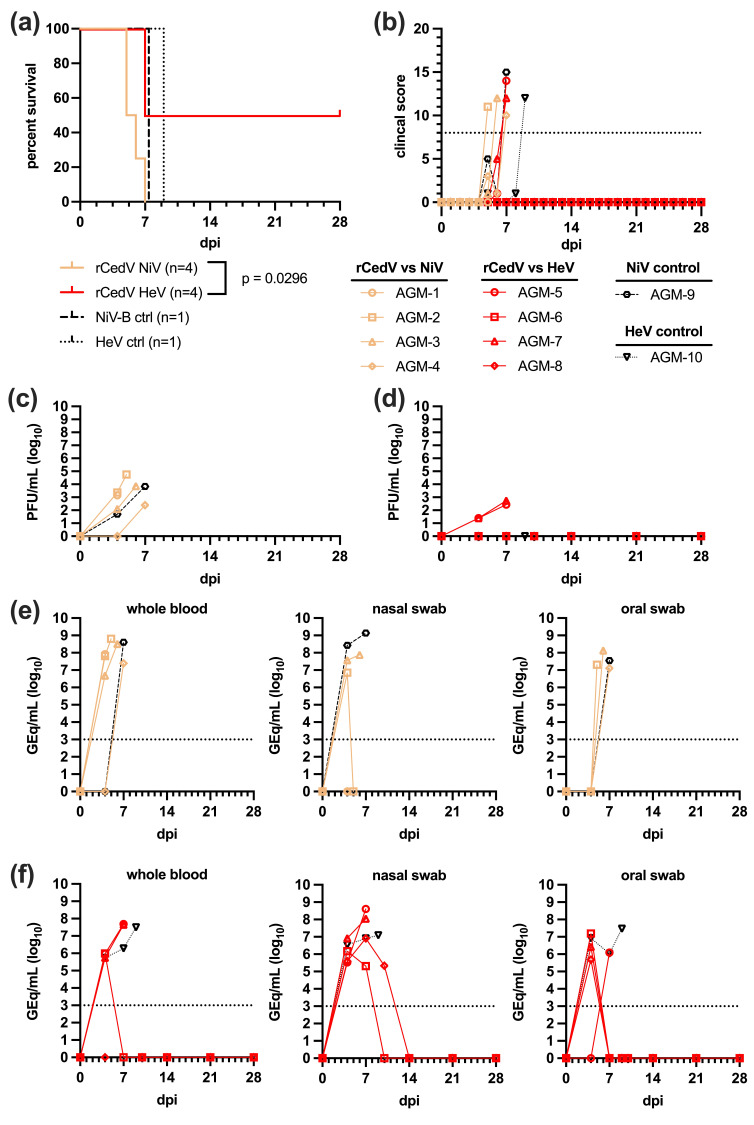
Survival, clinical scores, and viremia of rCedV infected AGMs challenged with NiV-B or HeV. (**a**) Kaplan–Meier survival curves of rCedV infected AGMs challenged with NiV-B or HeV compared with uninfected challenge controls. (**b**) Clinical scoring for rCedV infected AGMs challenged with NiV-B or HeV and their respective naïve controls. Each line represents each individual rCedV infected AGM challenged with NiV-B (AGM 1–4) or with HeV (AGM 5–8). As controls an AGM infected with NiV-B (AGM-9) only or with HeV (AGM-10) only are also represented. (**c**) Detection of NiV-B in plasma samples following back-challenge. (**d**) Detection of replicating HeV in plasma specimens following back-challenge. (**e**) NiV-B vRNA determined by qRT-PCR in blood, oral swabs, and nasal swabs, respectively. (**f**) HeV vRNA detected by qRT-PCR in whole blood, oral swabs, and nasal swabs, respectively. Dpi: days post-infection (from back-challenge).

AGMs 1–4 infected with NiV-B or AGMs 5–8 infected with HeV with a lethal dose (5.0E5 PFU) by an it/in combination route 42 days post-rCedV were monitored and assessed (Figure 5a,b). A single, naïve control AGM was included for each challenge group (NiV-B, AGM-9; HeV, AGM-10). Infection of AGMs 1–4 with NiV-B resulted in uniformly lethal outcomes by 7 dpi, and clinical signs were observed to be characteristic of severe NiV-B disease including weakness, dyspnea, unresponsiveness, and hypothermia (Supplementary Table S2). In the HeV cohort, 2/4 AGMs rapidly developed HeV disease and were euthanized at 7 dpi, while the two other subjects (AGM 6 and 8) survived to the end of the challenge study (day 28 post-HeV challenge), unlikely from prior rCedV infection and immune-based protection. When compared to the HeV control AGM (AGM-10), clinical signs observed were characteristic of HeV disease including weakness, recumbency, unresponsiveness, dyspnea, hypothermia, tachypnea, with only one subject (AGM-5) displaying nasal exudate (Supplementary Table S3). Both the NiV-B and HeV controls (AGM-9 and AGM-10, respectively) rapidly developed clinical disease consistent with henipavirus infection and were euthanized (Figure 5a). Clinical pathology assays indicated changes from baseline values following NiV-B challenge (Supplementary Table S2) or HeV challenge (Supplementary Table S3).

### 3.5. HeV and NiV Viremia and Viral Load in Back-Challenged AGMs

Following challenge with NiV-B and HeV, plasma specimens were collected at routine timepoints and analyzed for infectious virus by plaque assay. Replicating NiV-B was detected in 3/4 back-challenged AGMs starting at 4 dpi (Figure 5c), with titers ranging from 2.1 to 3.4 log10 PFU/mL. Peak viremia was observed at terminal timepoints and ranged from 2.4 to 4.8 log10 PFU/mL. The NiV-B control (AGM-9) had detectable viremia (1.7 log10 PFU/mL) at 4 dpi which peaked at 7 dpi (3.8 log10 PFU/mL). HeV was detected by plaque assay in plasma specimens of only 2/4 back-challenged AGMs and was not detected in the control subject at any point (Figure 5d). Replicating HeV was first detected at 4 dpi (1.4 log10 PFU/mL) and peaked at terminal timepoints (2.4–2.7 log10 PFU/mL).

Viremia detected by plaque assay correlated with qRT-PCR analysis of whole blood samples (Figure 5e). NiV-B vRNA was detected starting at 4 dpi (6.7–8.0 log10 GEq/mL) and peaked at clinical endpoints (7.4–8.8 log10 GEq/mL). NiV-B vRNA was detected only by 7 dpi in blood specimens collected from the control subject and AGM-4, which was the terminal timepoint (8.6 log10 GEq/mL). Nasal, oral, and rectal swabs were also collected throughout the course of the study for qRT-PCR analysis. NiV-B vRNA in nasal swabs was detected at 4 dpi in 2/4 AGMs (AGM 2 and 3) and the control subject (6.9–8.4 log10 GEq/mL) that increased at 7 dpi to 7.9 log10 GEq/mL for AGM-3 and 9.1 log10 GEq/mL for the control subject. AGM-2 did not have detectable NiV-B vRNA at 7 dpi. NiV-B vRNA was not detected in nasal swabs collected from AGM-1 or 4. Oral swabs collected from the cohort back-challenged with NiV-B had detectable vRNA in 3/4 AGMs; however, this was only observed at terminal timepoints (7.1–8.1 log10 GEq/mL). No rCedV, NiV-B, or HeV vRNA was detected in rectal swabs at any timepoint sampled.

HeV vRNA was detected by qRT-PCR in whole blood samples (Figure 5f) starting at 4 dpi (5.7–6.0 log10 GEq/mL) and peaked at terminal endpoints for the two AGMs with lethal outcomes (7.6–7.7 log10 GEq/mL). The control subject (AGM-10) had detectable vRNA in whole blood starting at 4 dpi (5.7 log10 GEq/mL), 7 dpi (6.3 log10 GEq/mL), and peaked at the clinical endpoint occurring 9 dpi (7.5 log10 GEq/mL). HeV vRNA was also detected in nasal swabs (Figure 5f) starting at 4 dpi (5.5–6.9 log10 GEq/mL) and peaked at terminal endpoints for AGM-5 and 7 (8.0–8.6 log10 GEq/mL). AGM-8 (survived to study endpoint) had HeV vRNA last detected in nasal swabs at 10 dpi (5.3 log10 GEq/mL). HeV vRNA was detected in the control subject (AGM-10) at all timepoints post-challenge with peak vRNA (7.1 log10 GEq/mL) observed at clinical endpoints. Oral swabs yielded similar results to nasal swabs in the HeV back-challenged cohort with vRNA quantities trending slightly lower and at fewer timepoints (Figure 5f). HeV vRNA was detected in oral swabs of 3/4 AGMs at 4 dpi (5.7–7.2 log10 GEq/mL). The HeV control subject (AGM-10) had peak vRNA detected in oral swabs at the clinical endpoint occurring 9 dpi (7.5 log10 GEq/mL).

### 3.6. Detection of NiV-B and HeV vRNA in Tissues of Back-Challenged AGMs

**Figure 6:**
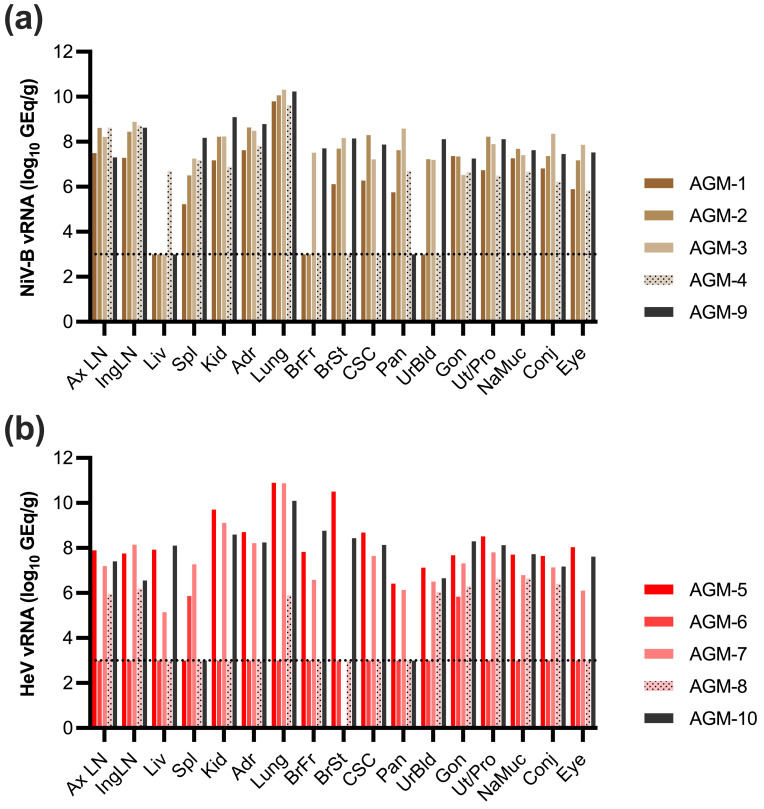
NiV-B or HeV viral load assessed by qRT-PCR in tissues of back-challenged AGMs. Detection of NiV-B vRNA (**a**) or HeV vRNA (**b**) in select tissues collected at necropsy. Each bar represents the mean of (2) technical replicates, and the horizontal line indicates the assay LOD (1000 GEq/g of tissues). Samples determined to be below the LOD are plotted at a value of 999 GEq/mL for visualization purposes. AxLN, axillary lymph node; IngLN, inguinal lymph node; Liv, liver; Spl, spleen; Kid, kidney; Adr, adrenal; BrFr, brain frontal; BrSt, brain stem; CSC, cervical spinal cord; Pan, pancreas, UrBld, urinary bladder; Gon, gonad; Ut/Pro, uterus/prostate; NaMuc, nasal mucosa; Conj, conjunctiva.

Tissues were collected at necropsy to detect levels of NiV-B and HeV vRNA by qRT-PCR (Figure 6). All subjects (AGM 1–4) back-challenged with NiV-B had detectable vRNA in all tissues assayed, with the highest quantities identified in the lung (9.6–10.3 log10 GEq/g), axillary lymph node (7.5–8.6 log10 GEq/g), inguinal lymph node (7.3–8.9 log10 GEq/g), and adrenal gland (7.6–8.6 log10 GEq/g) (Figure 6a). NiV-B vRNA was detected in other relevant tissues of henipavirus infection, including the spleen (5.2–7.3 log10 GEq/g) and kidney (6.9–8.2 log10 GEq/g). NiV-B vRNA was also detected in the CNS of 3/4 subjects challenged, with the greatest quantities identified in the brainstem and cervical spinal cord (6.1–8.2 log10 GEq/g and 6.3–8.3 log10 GEq/g, respectively). The NiV-B control subject (AGM-9) had equivalent levels of vRNA in all tissues, with the exception of kidney, where the vRNA level was identified to be 9.1 log10 GEq/g.

AGMs back-challenged with HeV that succumbed to disease (AGM-5 and 7) had the highest levels of HeV vRNA observed in the lungs (10.9 log10 GEq/g), kidney (9.1–9.7 log10 GEq/g), adrenal gland (8.2–8.7 log10 GEq/g), and 8.5–7.8 log10 GEq/g in the uterus/prostate (Figure 6b). Survivor subject AGM-6 only had detectable levels of vRNA in the spleen (5.9 log10 GEq/g) and gonad (5.8 log10 GEq/g). The other subject that survived to the study endpoint (AGM-8) had vRNA detected in several tissues; however, the highest levels were identified in the uterus/prostate (6.6 log10 GEq/g), nasal mucosa (6.6 log10 GEq/g), and conjunctiva (6.4 log10 GEq/g). The HeV challenge cohort in-study control, AGM-10, had detectable levels of vRNA in all tissues assayed except for spleen and pancreas. The highest levels of vRNA were identified in the lung (10.1 log10 GEq/g), brain frontal (8.8 log10 GEq/g), kidney (8.6 log10 GEq/g), and gonad (8.3 GEq/g).

### 3.7. Gross- and Histopathological Findings in AGMs Back-Challenged with NiV-B or HeV

**Figure 7:**
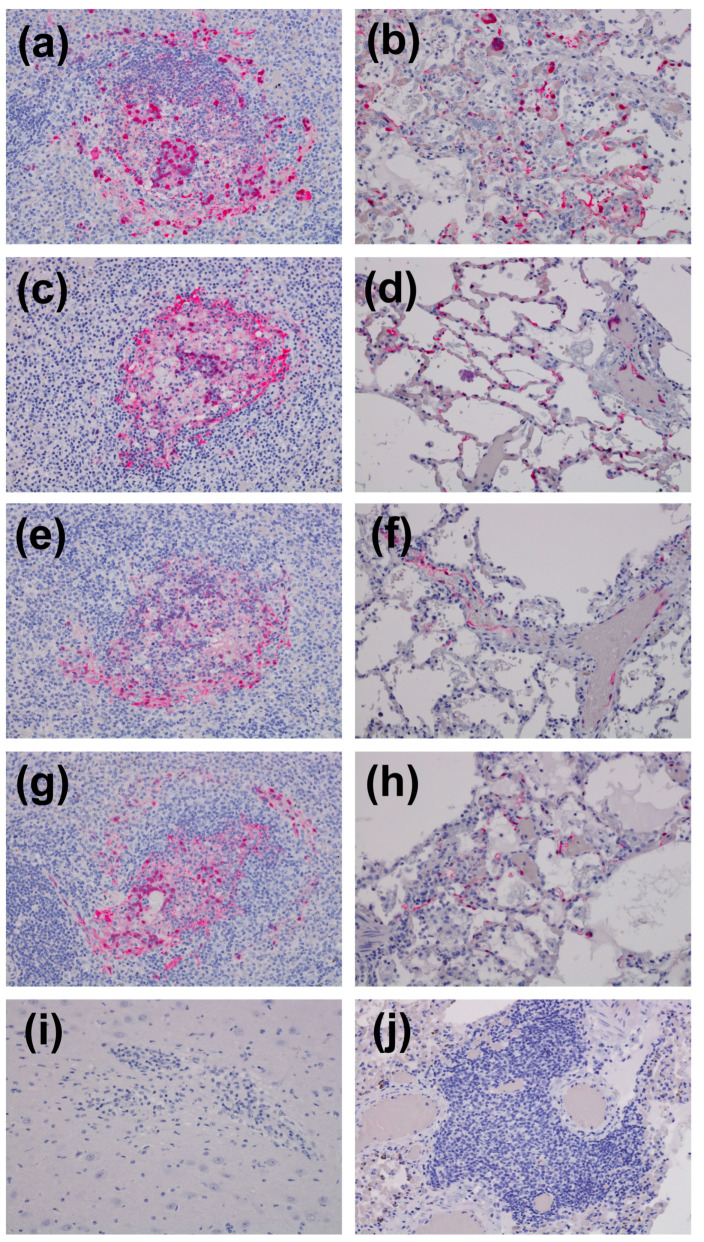
Representative immunohistochemistry images for anti-Henipavirus N antibody in rCedV infected AGMs challenged with NiV-B or HeV. Disruption of splenic follicles with IHC positive (red) mononuclear cells and syncytial cells in AGM-9 (**a**), AGM-2 (**c**), AGM-10 (**e**), and AGM-5 (**g**). Expansion of the pulmonary alveolar septa and flooding of alveolar spaces with IHC positive (red) mononuclear cells, endothelium, and the septal wall in AGM-9 (**b**), AGM-2 (**d**), AGM-10 (**f**), and AGM-5 (**h**). Perivascular lymphocytic infiltrates with an adjacent glial nodule in the brain (**i**) and perivascular lymphocytic infiltrate in the lung (**j**) of AGM-6 lacking IHC immunolabeling. All images captured at 20× magnification.

Microscopic evaluation of tissues collected from the back-challenge study included spleen, lung, and brain (frontal), brainstem/cerebellum, and hippocampus (Figure 7). Primary findings in lymphoid tissue included disruption of normal splenic follicle architecture characterized by necrosis, syncytial cell formation, and loss of lymphocytes. Positive immunohistochemistry (IHC) labeling for anti-henipavirus (N) antigen was noted in mononuclear cells and endothelial cells (Figure 7a,c,e,g). In the lungs, expansion of pulmonary alveolar septal walls with mixed inflammation and locally extensive areas of alveolar necrosis accompanied by fibrinaceous edema and increased numbers of alveolar macrophages were noted in all AGMs that succumbed to disease. Positive IHC labeling for henipavirus N antigen was noted in alveolar septa, mononuclear cells, and endothelial cells in the lung (Figure 7b,d,f,h). In the CNS tissue examined, lesions were not appreciated on H&E-stained sections in those that succumbed to disease; however, positive immunolabeling for henipavirus N antigen was identified in the gray matter of the cerebellum for AGM-5 and 7 (Supplementary Table S4). In the two surviving AGMs that were back-challenged with HeV-prototype, minimal lymphohistiocytic infiltrates were noted in the lung (AGM-6 and 8) and gliosis with rare glial nodule formation was observed in the brain of AGM-6. No positive immunolabeling was noted in any of the examined tissue sections of AGM-6 and AGM-8 (Figure 7i,j).

## 4. Discussion

The objective of this study was to characterize the pathogenicity of rCedV in AGMs and to determine if prior infection with rCedV afforded cross-protection against challenge with a lethal dose of either NiV-B or HeV. The pathogenicity of CedV has been investigated in experimental infections of ferrets and guinea pigs [14], hamsters [44] and albino IFNAR-KO mice [45], and here we report the first experimental infection of AGMs with rCedV.

In the studies described herein, we experimentally infected healthy, adult AGMs (n = 12) with a 5.0E5 PFU dose of rCedV via the it/in combined route; a uniformly lethal inoculum of NiV-B [56] and HeV-prototype. All rCedV-challenged subjects remained clinically well at 28 dpi (n = 4) and until back-challenge with NiV-B or HeV-prototype at 42 dpi (n = 8) (Figure 2). Similar survival trends were noted in ferrets (n = 2) and guinea pigs (n = 4) exposed to a higher dose of CedV (2.0E6 TCID50/mL) by the oronasal and intraperitoneal (ip) routes respectively [14]; Syrian golden hamsters inoculated ip or in with 1.0E5 TCID50 of CedV [44] and albino IFNAR-KO mice infected with 1.0E7 PFU of rCedV expressing a luciferase reporter (rCedV-Luc) via the ip route [45].

All rCedV infected AGMs developed a homologous neutralizing antibody response against CedV by day 28 post-infection. However, the induced immune response from rCedV infection did not result in a cross-neutralizing antibody response against NiV-B or HeV (Figure 3) and did not protect AGMs from lethal challenge with NiV-B (4/4) or HeV (2/4) (Figure 5). The induction of a homologous immune response to CedV infection was also noted in CedV infected ferrets and guinea pigs that developed an increasing antibody response against CedV with titers ranging from 1:320 to 1:1280 (ferrets) and 1:80 to 1:160 (guinea pigs) [14]. CedV infected Syrian golden hamsters and albino IFNAR-KO mice also developed neutralizing antibodies against CedV with titers ranging from ~1:40 to 1:640 [44] and a titer of ~1:1024 [45], respectively.

The P gene of HeV and NiV as well as the other emerging pathogenic henipaviruses produces the key interferon antagonists contributing to henipavirus pathogenicity. These antagonist proteins V and W are produced by the addition of non-templated G nucleotides in the editable RNA sequence of the P gene (reviewed in [47,48]). Genetic characterization revealed that the CedV P gene lacks not only RNA editing capability but also an open reading frame for the V and W proteins [14]. The lack of clinical disease and the subsequent neutralizing antibody response developed in rCedV infected AGMs observed in this study along with reported findings in guinea pigs, ferrets, Balb-C mice [14], hamsters [44] and albino C57BL/6 mice [45] further suggest that CedV displays a non-pathogenic phenotype in animal models of henipavirus infection; likely due to differences in the P gene.

Henipavirus infections result in severe and often fatal respiratory and/or neurological disease, and it is widely accepted that HeV and NiV utilize the highly conserved receptors, ephrin-B2 and ephrin-B3 [39,40,41]. Current understanding suggests that the neuronal tropism of NiV and HeV is linked to ephrin-B3 usage, and the respiratory tropism is linked to ephrin-B2 usage. Similar to HeV and NiV, CedV utilizes ephrin-B2 but not ephrin-B3, and instead uses additional ephrins-B1, -A2 and -A5 [43]. The differences in receptor usage and the lack of antagonist viral proteins could account for the lack of gross or histopathological evidence of overt henipavirus infection (namely vasculitis and/or syncytia) in the lung or brain of rCedV infected AGMs (AGMs 11, 12 and 14), despite the presence of mononuclear infiltrates in the lung of AGM-13 (Figure 4). These findings were in stark contrast to rCedV infected AGMs challenged with NiV-B (AGM 1–4) or HeV (AGM 5–8) and the respective controls (Figure 7).

In summary, we have confirmed in the NiV and HeV nonhuman primate model that CedV is nonpathogenic, providing important data in support of the classification of CedV as a Risk Group 2 (RG2) viral agent, thus providing a more accessible model system for researchers to employ. This classification will enable henipavirus researchers without access to a BSL-4 facility a sophisticated molecular toolkit to study henipavirus biology in a lower-containment setting. The study findings also reinforce the evidence that the ability of NiV and HeV to cause disease is linked to specific genetic factors such as the ability to produce the V and W interferon antagonist proteins. The limited survival of rCedV-challenged AGMs back-challenged with NiV-B or HeV also suggests that prior natural exposure to CedV will not offer protection against disease caused by pathogenic henipaviruses. Further, we have also developed and defined a new animal model using the AGM for the study of CedV infection and pathogenesis that underscores immunological differences between CedV and the pathogenic henipaviruses, which must be considered for the development of future henipavirus vaccine countermeasures.

## Data Availability

All study data are included in the main text or the Supplementary Materials of this manuscript.

## Supplementary Materials

The following supporting information can be downloaded at: https://www.mdpi.com/article/10.3390/v18030292/s1, Figure S1: Hematology and serum biochemistry of rCedV infected AGMs from 0 to 28 dpi; Table S1: Clinical Pathology of AGMs Following Challenge with Recombinant Cedar virus; Table S2: Clinical Pathology of rCedV challenged AGMs Following Back-Challenge with Nipah virus; Table S3: Clinical Pathology of rCedV challenged AGMs Following Back-Challenge with Hendra virus; Table S4: H&E and IHC Severity Scores of AGMs back-challenged with NiV-B or HeV-prototype.
