# Supplementary material for: Analysis of Recombinant Cedar Virus Infection and Cross-Protection Against Related Henipaviruses in African Green Monkeys
Source: Viruses. 2026 Feb 28;18(3):292. doi: 10.3390/v18030292 (PMC13030515; doi:10.3390/v18030292)
Supplement: Supplementary file 1 [file viruses-18-00292-s001.zip › viruses-4137903-supplementary.pdf]

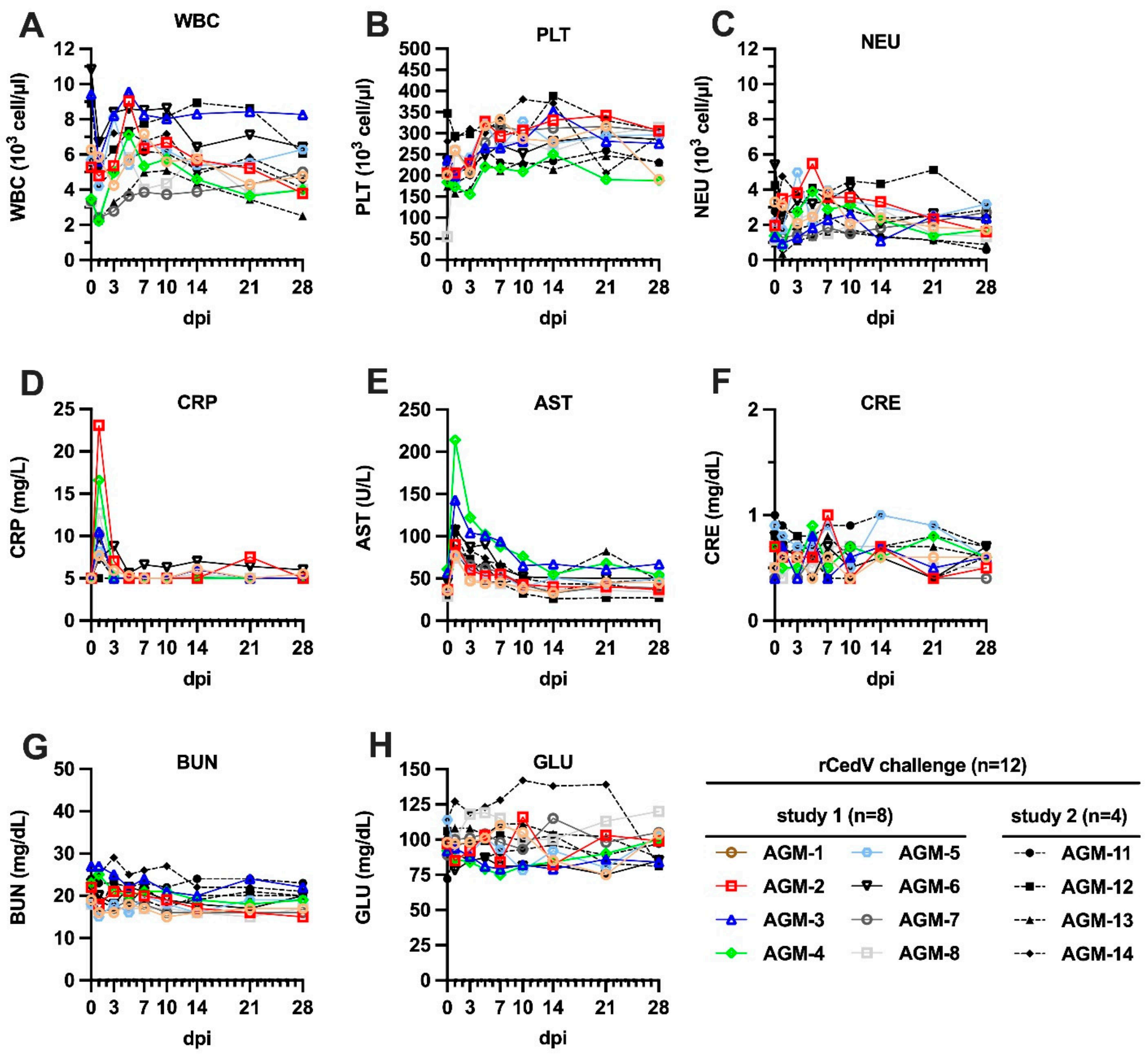

Supplementary Figure S1: Hematology and serum biochemistry of rCedV infected AGMs from 0 to 28 dpi. WBC, white blood cells; PLT, platelets; NEU, neutrophils; CRP, c-reactive protein; AST, aspartate transferase; CRE, creatinine; BUN, blood urea nitrogen; GLU, glucose; dpi, days post infection.

Supplementary Table S1: Clinical Pathology of AGMs Following Challenge with Recombinant Cedar virus.

Subject No.	Sex	Clinical Illness*	Clinical Pathology*
AGM-1	Female	None. Subject survived to study endpoint (d28).	↑AST (d1);
AGM-2	Female	None. Subject survived to study endpoint (d28).	Hyperglycemia (d5); ↓BUN (d5); hypocalcemia (d5); hypoalbuminemia (d5); hypoproteinemia (d5); ↑AST (d1); hypoamylasemia (d5); ↑CRP (d1).
AGM-3	Male	None. Subject survived to study endpoint (d28).	↑CRE (d1); ↑AST (d1); ↑CRP (d1).
AGM-4	Male	None. Subject survived to study endpoint (d28).	↑AST (d1, 3); ↑CRP (d1).
AGM-5	Male	None. Subject survived to study endpoint (d28).	Hypoglycemia (d10, 21); ↑AST (d1).
AGM-6	Male	None. Subject survived to study endpoint (d28).	↓CRE (d5, 21); ↑AST (d1).
AGM-7	Female	None. Subject survived to study endpoint (d28).	↑AST (d1); ↑CRP (d1).
AGM-8	Female	None. Subject survived to study endpoint (d28).	↑AST (d1); ↑CRP (d1).
AGM-11	Male	None. Subject survived to study endpoint (d28).	Monocytopenia (d1, 10); monocytosis (d21); neutropenia (d1, 3, 10, 14, 21, 28); eosinopenia (d3, 14, 28); basopenia (d1, 3, 28); ↑AST (d1).
AGM-12	Female	None. Subject survived to study endpoint (d28).	Lymphocytopenia (d1, 28); monocytopenia (d1, 7, 21); neutropenia (d1, 3); eosinopenia (d1, 3, 5); basopenia (d1, 3); ↓CRE (d7, 10, 21); ↑AST d1, 3).
AGM-13	Male	None. Subject survived to study endpoint (d28).	Lymphocytopenia (d28); monocytopenia (d1, 3, 10, 14, 21); neutropenia (d1); eosinopenia (d1, 3, 5, 7); basopenia (d1, 3); ↑ALT (d21); ↑AST (d1, 21).
AGM-14	Female	None. Subject survived to study endpoint (d28).	Lymphocytopenia (d28); monocytopenia (d21); monocytosis (d3, 5, 10, 14); neutrophilia (d1); eosinopenia (d21); basopenia (d21); hypoglycemia (d28); ↑AST (d1, 3).

*Days after rCedV challenge in parentheses. All reported findings are in comparison to baseline (day of challenge, i.e. d-0) values. Hyperglycemia is defined as a 100% or greater increase in levels of glucose. Hypoglycemia is defined by a ≥25% decrease in levels of glucose. Hypoalbuminemia is defined by a ≥25% decrease in levels of albumin. Hypoproteinemia is defined by a ≥25% decrease in levels of total protein. Hypoamylasemia is defined by a ≥25% decrease in levels of serum amylase. Hypocalcemia is defined by a ≥25% decrease in levels of serum calcium. Lymphocytopenia, monocytopenia, neutropenia, eosinopenia, and basopenia are defined by a ≥35% drop in numbers of lymphocytes, monocytes, neutrophils, eosinophils, or basophils, respectively. Monocytosis and neutrophilia are defined by a 100% or greater increase in numbers of monocytes and neutrophils, respectively. Increases in AST, BUN, CRE and CRP were graded on the following scale: ↑ = 1-5-fold, ↑↑ = >5-10 fold, ↑↑↑ = >10-20 fold, ↑↑↑↑ = >20-fold, ↓ = ≥50% decrease. ALT, alanine transaminase; AST, aspartate aminotransferase; BUN, blood urea nitrogen; CRE, Creatinine; CRP, C-reactive protein.

Supplementary Table S2: Clinical Pathology of rCedV challenged AGMs Following Back-Challenge with Nipah virus.

Subject No.	Sex	Clinical Illness*	Clinical Pathology*
AGM-1	Female	Subject euthanized (d5).	Lymphocytopenia (d4); ↑CRP (d4).
AGM-2	Female	Depression (d5); decreased appetite (d5); weakness (d5); recumbency (d5); unresponsiveness (d5); dyspnea (d5). hypothermia (d5). Subject euthanized (d5).	Thrombocytopenia (d5); lymphocytopenia (d4); monocytosis (d5); neutrophilia (d5); eosinophilia (d4, 5); basophilia (d4); hyperglycemia (d5); ↑BUN (d5); ↑CRE (d5); hypoalbuminemia (d5); hypoproteinemia (d5); ↑AST (d5); ↑CRP (d5).
AGM-3	Male	Depression (d6); decreased appetite (d1, 3, 4, 5, 6); weakness (d6); recumbency (d6); dyspnea (d6); serosanguineous oronasal exudate (d6); hypothermia (d6). Subject euthanized (d6).	Thrombocytopenia (d6); lymphocytopenia (d6); neutrophilia (d6); eosinophilia (d6); basopenia (d4); hyperglycemia (d6); ↑CRE (d6); hypoalbuminemia (d6); ↑AST (d6); hypoamylasemia (d6); ↑CRP (d4); ↑↑↑CRP (d6).
AGM-4	Male	Depression (d7); decreased appetite (d1, 3-7); weakness (d7); recumbency (d7); hunched posture (d7); dyspnea (d7); tachypnea (d7). Subject euthanized (d7).	Thrombocytopenia (d7); lymphocytopenia (d7); monocytosis (d4); monocytopenia (d7); neutropenia (d4); neutrophilia (d7); eosinopenia (d4); eosinophilia (d7); basophilia (d7); ↑AST (d7); hypoamylasemia (d7); ↑CRP (d7).
AGM-9	Male	Decreased appetite (d1-7); weakness (d7); recumbency (d7); unresponsiveness (d7); dyspnea (d7), tachypnea (d7); hypothermia (d7). Subject euthanized (d7).	Lymphocytopenia (d7); monocytopenia (d4, 7); neutrophilia (d7); eosinophilia (d4); basophilia (d7); ↑CRE (d7); hypoalbuminemia (d7); hypoamylasemia (d7); ↑CRP (d7).

*Days after NiV-B challenge in parentheses. All reported findings are in comparison to baseline (day of challenge, i.e. d-0) values. Decreased appetite is defined as some food but not all food consumed from the previous day. Anorexia is defined as no food consumed from the previous day. Fever is defined as a temperature more than 2.5 °F over baseline, or at least 1.5 °F over baseline and ≥ 103.5 °F. Hypothermia is defined as a temperature ≤3.5°F below baseline. Lymphocytopenia, monocytopenia, erythrocytopenia, thrombocytopenia, neutropenia, eosinopenia, and basopenia are defined by a ≥35% drop in numbers of lymphocytes, monocytes, erythrocytes, platelets, neutrophils, eosinophils, or basophils, respectively. Lymphocytosis, monocytosis, neutrophilia, eosinophila, and basophilia are defined by a 100% or greater increase in numbers of lymphocytes, monocytes, neutrophils, eosinophils, and basophils, respectively. Hyperglycemia is defined as a 100% or greater increase in levels of glucose. Hypoglycemia is defined by a ≥25% decrease in levels of glucose. Anemia is defined as a concurrent ≥25% decrease in erythrocyte count, HCT, and HGB. Hypoalbuminemia is defined by a ≥25% decrease in levels of albumin. Hypoproteinemia is defined by a ≥25% decrease in levels of total protein. Hypoamylasemia is defined by a ≥25% decrease in levels of serum amylase. Hypocalcemia is defined by a ≥25% decrease in levels of serum calcium. Increases in ALT, AST, ALP, CRE, CRP, HCT, and HGB were graded on the following scale: ↑ = 1-5-fold, ↑↑ = >5-10 fold, ↑↑↑ = >10-20 fold, ↑↑↑↑ = >20-fold, ↓ = ≥50% decrease. (BUN) blood urea nitrogen, (ALT) alanine aminotransferase, (AST) aspartate aminotransferase, (ALP) alkaline phosphatase, (CRE) Creatinine, (CRP) C-reactive protein, (HCT) hematocrit, (HGB) hemoglobin.

Supplementary Table S3: Clinical Pathology of rCedV challenged AGMs Following Back-Challenge with Hendra virus.

Subject No.	Sex	Clinical Illness*	Clinical Pathology*
AGM-5	Male	Decreased appetite (d0-7); weakness (d7); recumbency (d7); unresponsiveness (d7); dyspnea (d7); tachypnea (d7); nasal exudate (d7). Subject euthanized (d7).	Thrombocytopenia (d7); monocytosis (d7); neutropenia (d4); neutrophilia (d7); eosinophilia (d7); basopenia (d7); hypoalbuminemia (d7); ↑CRP (d4).
AGM-6	Male	Decreased appetite (d3-12, 15). Subject survived to study endpoint (d28).	Thrombocytopenia (d7); lymphocytosis (d4, 7, 10, 14, 21, 28); monocytosis (d10, 14); neutropenia (d10, 28); eosinophilia (d14, 21, 28); basopenia (d7); basophilia (d10, 14, 21); ↓CRE (d28); ↑CRP (d4).
AGM-7	Female	Decreased appetite (d6, 7); depression (d7); weakness (d7); recumbency (d7); dyspnea (d7); tachypnea (d7); hypothermia (d7). Subject euthanized (d7).	Thrombocytopenia (d7); monocytosis (d4); neutropenia (d4); neutrophilia (d7); eosinopenia (d4); hyperglycemia (d7); ↑BUN (d7); ↑CRE (d7); hypoproteinemia (d7); ↑CRP (d4, 7).
AGM-8	Female	Decreased appetite (d3, 5-10). Subject survived to study endpoint (d28).	Lymphocytopenia (d10, 14, 21, 28); monocytopenia (d7, 10, 21); neutropenia (d4, 7, 10, 14, 21, 28); eosinopenia (d4, 7, 10, 14, 21, 28); basopenia (d4, 7, 10, 14, 21, 28); ↓CRE (d7, 14); ↑CRP (d4).
AGM-10	Female	Depression (d9); decreased appetite (d5-9); weakness (d9); recumbency (d9); unresponsiveness (d9); dyspnea (d9); hypothermia (d9). Subject euthanized (d9).	Thrombocytopenia (d9); lymphocytopenia (d7); monocytosis (d4, 9); neutropenia (d4); eosinophilia (d9); basopenia (d4); basophilia (d9); hyperglycemia (d9); ↑CRE (d9); hypoalbuminemia (d9); hypoamylasemia (d9); ↑CRP (d7).

*Days after HeV challenge in parentheses. All reported findings are in comparison to baseline (day of challenge, i.e. d-0) values. Decreased appetite is defined as some food but not all food consumed from the previous day. Anorexia is defined as no food consumed from the previous day. Fever is defined as a temperature more than 2.5 °F over baseline, or at least 1.5 °F over baseline and ≥ 103.5 °F. Hypothermia is defined as a temperature ≤3.5°F below baseline. Lymphocytopenia, monocytopenia, erythrocytopenia, thrombocytopenia, neutropenia, eosinopenia, and basopenia are defined by a ≥35% drop in numbers of lymphocytes, monocytes, erythrocytes, platelets, neutrophils, eosinophils, or basophils, respectively. Lymphocytosis, monocytosis, neutrophilia, eosinophila, and basophilia are defined by a 100% or greater increase in numbers of lymphocytes, monocytes, neutrophils, eosinophils, and basophils, respectively. Hyperglycemia is defined as a 100% or greater increase in levels of glucose. Hypoglycemia is defined by a ≥25% decrease in levels of glucose. Anemia is defined as a concurrent ≥25% decrease in erythrocyte count, HCT, and HGB. Hypoalbuminemia is defined by a ≥25% decrease in levels of albumin. Hypoproteinemia is defined by a ≥25% decrease in levels of total protein. Hypoamylasemia is defined by a ≥25% decrease in levels of serum amylase. Hypocalcemia is defined by a ≥25% decrease in levels of serum calcium. Increases in ALT, AST, ALP, CRE, CRP, HCT, and HGB were graded on the following scale: ↑ = 1-5-fold, ↑↑ = >5-10 fold, ↑↑↑ = >10-20 fold, ↑↑↑↑ = >20-fold, ↓ = ≥50% decrease. (BUN) blood urea nitrogen, (ALT) alanine aminotransferase, (AST) aspartate aminotransferase, (ALP) alkaline phosphatase, (CRE) Creatinine, (CRP) C-reactive protein, (HCT) hematocrit, (HGB) hemoglobin.

Table S4: H&E and IHC Severity Scores of AGMs back-challenged with NiV-B or HeV-prototype.

Animal ID	AGM-9	AGM-1	AGM-2	AGM-3	AGM-4	AGM-10	AGM-5	AGM-7	AGM-6	AGM-8
	CTL	Back challenged with Nipah-Bangladesh				CTL	Back challenged with Hendra-prototype			
dpi	7	5	5	6	7	9	7	7	28-depop	28-depop
Spleen	2 2	2 2	2 2	2 2	2 1	2 1	2 2	2 2	0 0	0 0
Lung	2 2	2 2	2 2	2 2	2 1	2 2	3 2	2 1	1 0	1 0
Brain (Frontal)	0 0	0 0	0 0	0 0	0 0	0 0	0 0	0 0	0 0	0 0
Brainstem/Cerebellum	0 0	0 0	0 0	0 0	0 0	0 0	0 2	0 2	1 0	0 0
Brain (Hippocampus)	0 0	0 0	0 0	0 0	0 0	0 0	0 0	0 0	1 0	0 0

First score indicates severity score of H&E slide
Second score indicates severity score of IHC slide
0 = No lesions
1 = 1-25% of examined tissues with lesions or IHC labeling, 1 cell type that is IHC positive
2 = 26-50% of examined tissues with lesions or IHC labeling, up to 2 cell types that are IHC positive
3 = 51-75% of examined tissues with lesions or IHC labeling, up to 2 cell types that are IHC positive
4 = 76-100% of examined tissues with lesions or IHC labeling, up to 2 cell types that are IHC positive
